# Design and Optimization of a Biosensor Surface Functionalization to Effectively Capture Urinary Extracellular Vesicles

**DOI:** 10.3390/molecules26164764

**Published:** 2021-08-06

**Authors:** Agnieszka Kamińska, Magdalena E. Marzec, Ewa Ł. Stępień

**Affiliations:** Department of Medical Physics, Marian Smoluchowski Institute of Physics, Faculty of Physics, Astronomy and Applied Computer Science, Jagiellonian University, 30-348 Kraków, Poland; agnieszka1.kaminska@uj.edu.pl (A.K.); e.stepien@uj.edu.pl (E.Ł.S.)

**Keywords:** extracellular vesicles, lactadherin, ToF-SIMS

## Abstract

For this study, we tested and optimized silicon surface functionalization procedures for capturing urinary extracellular vesicles (uEVs). The influence of the silane type (APTES or GOPS) and protein concentration on the efficiency of uEVs binding was investigated. Human lactadherin protein (LACT) was used to capture uEVs. We applied surface characterization techniques, including ellipsometry, atomic force microscopy, and time-of-flight secondary ion mass spectrometry, to observe changes in the biosensor surface after each functionalization step. uEVs were purified by a low-vacuum filtration method and concentrated by ultracentrifugation. The physical parameters of uEVs after the isolation procedure, such as morphology and size distribution, were determined using transmission electron microscopy and tunable resistive pulse sensing methods. We observed a gradual growth of the molecular layer after subsequent stages of modification of the silicon surface. The ToF-SIMS results showed no changes in the mean intensities for the characteristic peaks of amino acids and lipids in positive and negative polarization, in terms of the surface-modifying silane (APTES or GOPS) used. The most optimal concentration of LACT for the tested system was 25 µg/mL.

## 1. Introduction

The demand for fast and inexpensive diagnostic methods to enable the screening or control of early symptoms of renal insufficiency, on an outpatient basis, has been invariably growing for many years [1,2]. The application of biological recognition, based on the design of a biosensor (i.e., a selective, highly sensitive system analyzing selected substances at the molecular level), is a breakthrough which provides hope for diagnostics [3]. The development of an analytical device to determine the presence of specific substances in the tested sample requires thoughtful design and meticulous implementation. Each stage of preparation is important and affects the operation of the future signal converter [4]. The basis of the operation of biosensors—in their biological part—is the capture of specific molecules in the studied environment, such as chemical compounds, proteins, and even micro-organisms (e.g., bacteria, prions, and viruses), through the use of chemical, immunological, or biocatalytic ligands [5].

There are many methods for the immobilization of receptors on a biosensor surface, consisting of physical or chemical bonding with the substrate [6]. Often, covalent binding with the receptor takes place through self-assembling monolayers (SAM), thus changing the surface of the sensor [7]. The functional groups of SAM compounds on the surface of biosensors and the functional groups containing the deposited receptor determine the course and effectiveness of immobilization of the receptor on the surface [6,8].

Equally important is the final immobilization stage (i.e., carrying out the binding reaction of bioactive substances), which often involves highly absorbent or fluorescent dyes, through the use of a monolayer and terminal substituents. At the same time, in order to optimize the work of biosensors, an important aspect is to minimize non-specific interactions and increase the surface-related biological activity of the probe, through careful design of the orientation, conformation, and concentration of the biomolecule [6].

The proper distribution of receptor molecules on the functionalized surface is key in ensuring the efficiency of a biosensor [9]. It is important to maximize the use of the space, in order to achieve high miniaturization of the device, as well as to effectively cover the available space with the binding centers to appropriately use the available material.

The spatial structure of the captured molecule plays an equally important role, due to steric effects, which often make it impossible to capture the molecule of interest. It should be remembered that the size of the molecule and its spatial distribution play important roles in the design of biosensors, as they are associated with the blocking of other binding sites, through use of the bound molecule [10].

Thus, preparing a homogeneous detection substrate is a multi-stage, challenging process that involves great experimental difficulties. To date, there have been many successful examples of the production of specialized biosensors, such as those for prostate-specific antigen capture [11], detecting DNA strands, or a commercially available blood glucose sensor [12].

Extracellular vesicles (EVs) are intensively studied biological structures, of which more and more have become known recently and whose diagnostic importance is rapidly expanding. It is a heterogeneous population of spherical structures that are naturally released by cells and circulate in body fluids (e.g., urine). These structures are characterized by unique properties that endow them with great diagnostic and therapeutic potential. They are often transporters of bioactive molecules, such as proteins, nucleic acids, lipids, or metabolites, from parental to target cells, making them mediators of intercellular communication [13]. As the molecular composition of EVs may reflect the state of the cell, they can act as disease biomarkers [14,15]. Lab-on-a-chip methods have gained more and more recognition in the field of EV isolation and detection in recent years [16,17]. At present, methods based on microfluidic systems, which enable the simultaneous automatic purification of samples, are of particular importance [18].

In this study, human recombinant lactadherin (LACT), also known as MFG-E8, is applied as the EV recognition layer. This small glycoprotein (46 kDa) comprises three domains: Epidermal growth factor (EGF-like), which has an affinity for the αvβ3 and αvβ5 integrins; the C2 domain that binds to phosphatidylserine (PS); and the C1 domain. It was first isolated from cow milk fat globules but has also been found in other tissues and organs, including the mammary gland and epididymis epithelium, biliary cells, body fluids, and sweat glands [19,20,21]. LACT has an affinity to components present on the surface of the EV membrane, such as phosphatidylserine (PS) and integrin αvβ3 [22]. LACT is a component of milk fat globules and contains an analogous epidermal growth factor (EGF) domain at the N-terminus and two C-discoidin-like lectin domains, similar to the phosphatidylserine (PS)-binding domains of coagulation factors V and VIII [23]. Thus, the EGF LACT domain contains an RGD adhesion motif which is recognized by integrin αvβ3. The C2 domain has an affinity for PS [22]. The major advantage of LACT is that the binding process of EVs with this protein is not dependent on the presence of Ca^2+^ ions, which are required for annexin V binding [23,24]. The new potential of LACT in development of the delivery systems PS-exposing enveloped viruses has been proposed [19].

The aim of the presented work is to develop and optimize the procedure of functionalization of the silicon surface for the capture of urinary extracellular vesicles (uEVs). This article focuses on two important aspects of uEV recognition by a LACT-based sensor: (i) The contribution of the silane used to the binding efficacy of the LACT protein; and (ii) the effect of the protein concentration on uEV capture. To solve these issues, two different silanes were tested to functionalize the surface: 3-aminopropyltriethoxysilane (APTES) and 3-glycidyloxypropyltrimethoxysilane (GOPS). Further, three LACT concentrations were applied: 25, 50, and 100 µg/mL.

The physical characterization of EVs was performed using two techniques: transmission electron microscopy (TEM) and tunable resistive pulse sensing technology (TRPS). The efficiency of operation—and, thus, the capture of EVs by the prepared surfaces—was examined by three physical methods: spectroscopic ellipsometry (SE), atomic force microscopy (AFM), and time-of-flight mass spectrometry (ToF-SIMS). There exist several research examples showing that the methods used are complementary to each other and allow for a comprehensive view of the examined surface [25,26].

## 2. Results and Discussion

### 2.1. Characterization of the Size Distribution and Morphology of uEVs

Two methods were used to characterize uEVs: (i) Transmission electron microscopy (TEM), in order to characterize the size, morphology, and observe the diversity in molecular cargo of uEVs; and (ii) tunable resistive pulse sensing (TRPS), in order to determine the uEVs size distribution from the condensed urine sample. 

**Figure 1:**
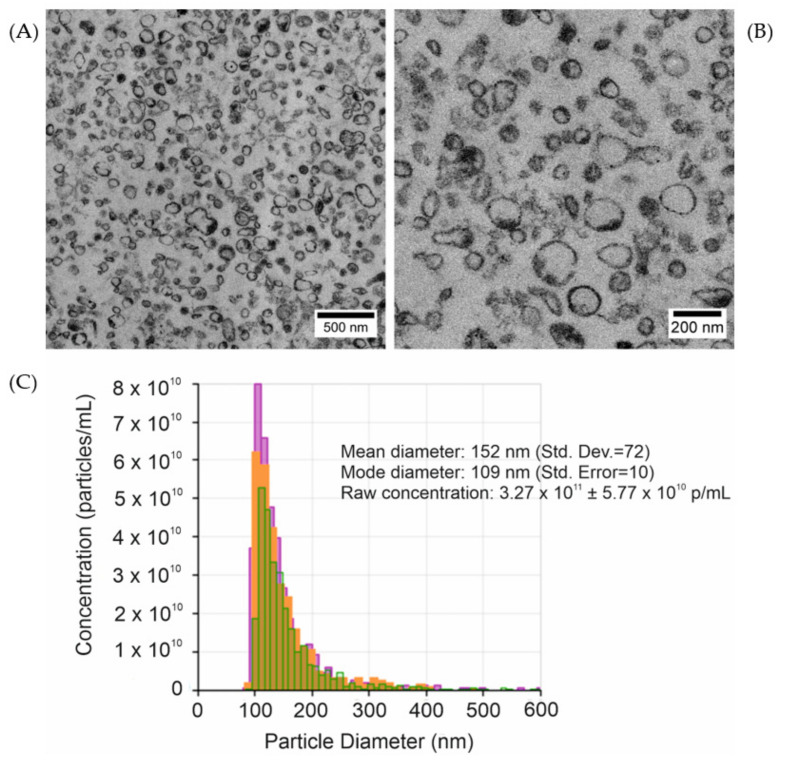
(**A**,**B**) Transmission electron micrographs of isolated uEVs at two different magnifications; and (**C**) the size distribution of uEVs obtained by qNano system.

TEM analysis confirmed the presence and integrity of uEVs in the sample after isolation. Representative TEM images of uEVs are shown in Figure 1A,B. TEM micrographs demonstrated the heterogeneity of uEVs, with regard to size, morphology, and electron density. The size distribution of uEVs obtained by TRPS technology revealed that most of the uEVs were between 100 and 300 nm, representing medium and large EVs (Figure 1C) [27].

### 2.2. Surface Characterization

Three physical techniques were used to characterize each step of biomolecule immobilization: (i) Spectrometric ellipsometry (SE), to calculate the thickness of the molecular layer formed on the surface after each modification step; (ii) atomic force microscopy (AFM), for observation of the surface topography and calculation of the surface roughness; and (iii) ToF-SIMS, for the detection of LACT molecules and uEVs on a prepared surface.

#### 2.2.1. Estimating the Thickness of the Biomolecular Layer Based on Ellipsometry Measurements

Thickness resolution and in situ information provided by ellipsometry make this optical method particularly suitable for the study of thin organic layers [28]. For this reason, after each preparation stage, ellipsometry was used to determine the thickness of the deposited molecular layer on the tested substrate. This approach allows for the evaluation of the next stages of preparation, and to determine which of the approaches used provides the best solution for obtaining the most functional surface. 

The tested layers in this work had a low refractive index and their typical thicknesses were in the nanometer range; therefore, in order to resolve the optical limitations and observe microstructural details, thus going beyond simple layer detection, careful selection of the experimental and analytical methods should be carried out. 

In the first step, APTES-modified substrates functionalized with aldehyde groups (GA) were measured, in order to prepare the surface for protein binding. The initial step of surface functionalization in the first experiment was the production of a thin, stable silane layer using APTES. The thickness of obtained layer was 1.2 ± 0.4 nm. The second step of immobilizing the biomolecules was to treat the surface of the silane layer with GA. This compound is a homobifunctional crosslinker, with aldehyde groups at both ends having a carbon chain spacer. Surface modification with APTES + GA resulted in the production of the silane layer with a thickness of 2.1 ± 0.1 nm on silicon, which was close to the corresponding values reported in the literature [28]. With GOPS, a single-particle silane layer with a thickness of 1.5 ± 0.1 nm was obtained.

In the next step, the LACT protein was applied to all of the prepared surfaces. For the ellipsometric measurements, only one LACT concentration (50 µg/mL) was used. The application of the protein caused a visible change in the thickness of the deposited layer. The thickness of the LACT layer on the APTES and GOPS substrate was equal to 9 ± 1 nm. Finally, the uEV sample was applied to the protein, which enabled the formation of a layer of 37 ± 9 nm for GOPS and 22 ± 2 nm for APTES, respectively. SE is a relatively fast, accurate, and sensitive method to observe even the slightest changes on the surface of a solid. These advantages enabled effective characterization of the subsequent stages of preparation of the biosensor under development.

#### 2.2.2. AFM Imaging

**Figure 2:**
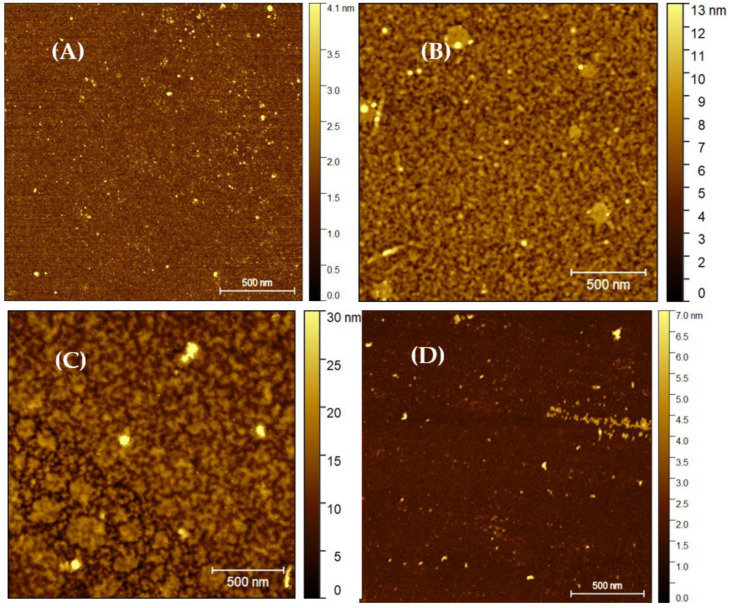
Non-contact AFM images: (**A**) raw silicon surface; (**B**) substrate functionalized with APTES; (**C**) thin film ended with GA on APTES; and (**D**) substrate subjected to silanization with GOPS. The size of the scanning area is 2 × 2 μm^2^.

In this work, dried samples on a silicon substrate were imaged using AFM in the non-contact mode. In this mode of operation, the cantilever vibrates at a natural resonant frequency and with a low amplitude above the sample surface. As the cantilever approaches the surface, the forces of attraction and repulsion modify the resonant frequency of the cantilever, which is coupled to the surface. Controlling this frequency allows for adjustment of the tip–sample distance. Then, it becomes possible to map the sample surface. Figure 2 shows the maps after each sample preparation stage and before the LACT loading stage.

The Sq RMS roughness result for the bare silicone substrate (Figure 2A) was 0.73 nm. In the next step, the surface roughness significantly increased with the silanization process, resulting in measured Sq RMS values of 1.72 nm for APTES (Figure 2B) and 1.26 nm for GOPS (Figure 2D). This can be explained by the formation of a multi-layer, thin, and stable silane layer. In the case of Figure 2C, chemical ligation of GA to the amino terminated surface resulted in the Sq RMS value of 1.21 nm. This observation indicated that the treatment with GA resulted in a more uniform surface topography, compared to the APTES termination.

**Figure 3:**
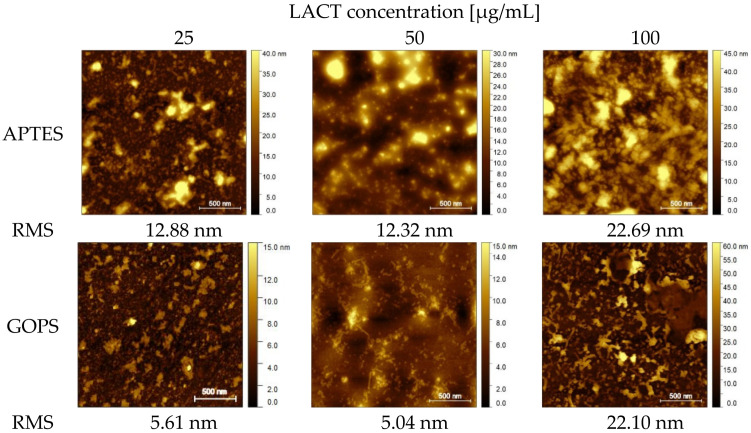
Non-contact AFM images for substrates subjected to silanization with APTES/GA+LACT and GOPS+LACT, on which the protein was applied at specific concentrations. The size of scanning area is 2 × 2 μm^2^.

As can be seen in Figure 3, the Sq RMS values significantly increased for both types of silanes. For APTES, with the two lower concentrations, the values were 12.88 nm and 12.32 nm, corresponding to the concentrations of 25 and 50 µg/mL, respectively. In the case of 100 µg/mL concentration, an almost two-fold increase in roughness can be noticed, as the Sq RMS parameter had a value of 22.69 nm. An analogous increase in the Sq RMS value for the GOPS silane can be observed. For this surface functionalization, for the two lower protein concentrations, the roughness values for the 25 µg/mL concentration increased to 5.61 nm, while that for 50 µg/mL increased to 5.04 nm. With the highest concentration (100 µg/mL), the Sq RMS value was 22.10 nm. For both types of silanes, it can be seen that the highest roughness characterized the samples to which the above protein concentrations were applied; however, the lowest values were obtained for the concentration of 50 µg/mL for both APTES and GOPS.

**Figure 4:**
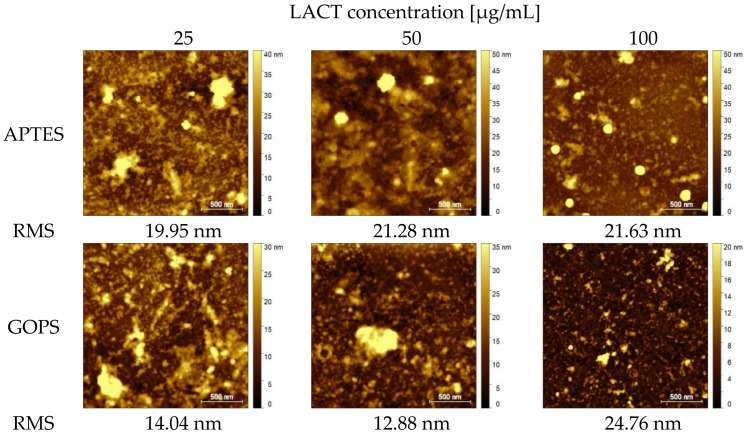
Non-contact AFM images for substrates silanized with APTES and GOPS, onto which LACT protein (25, 50, 100 µg/mL) and urine extracellular vesicles were applied. The size of scanning area is 2 × 2 μm^2^.

The AFM images obtained for the surfaces on which the uEVs were applied are summarized in Figure 4. In Figure 4, two types of silanes and three concentrations of LACT protein on which the uEVs were applied are presented. For samples with uEVs, an increase in Sq RMS surface roughness was observed for the first two concentrations for both silanes. Meanwhile, for APTES, for the concentration of 25 µg/mL, the Sq RMS value was 19.95 nm; for the concentration of 50 µg/mL, it was 21.28 nm; and, for 100 µg/mL, the roughness value slightly decreased. The values for the second type of silane, GOPS, were 14.04 nm at 25 µg/mL, 12.88 nm at 50 µg/mL, and 24.76 nm for the highest concentration (100 µg/mL).

#### 2.2.3. ToF-SIMS Analysis

ToF-SIMS was used for a comparative analysis, in order to diagnose which of the approaches used in the preparation were the best. In this experiment, we checked which surface functionalization led to the highest efficiency of uEVs capture.

For this purpose, each surface was analyzed in the spectrometric mode. Such a test provides detailed information about the chemical composition, without risk of damaging the surface. For comparative analysis, all measurements were made under the same conditions and in one experiment, carried out at the same time. All spectra obtained were scaled to total counts.

As stated in the introduction, LACT is a molecule used for the capture of uEVs. The optimal deposition of this protein on the surface generates a better efficiency in capturing of EVs in the following step. For this reason, the first stage of the comparative analysis focused on determining which preparation approach generates the highest intensities for the characteristic peaks of the amino acids which are the building blocks of the protein.

LACT Binding

**Figure 5:**
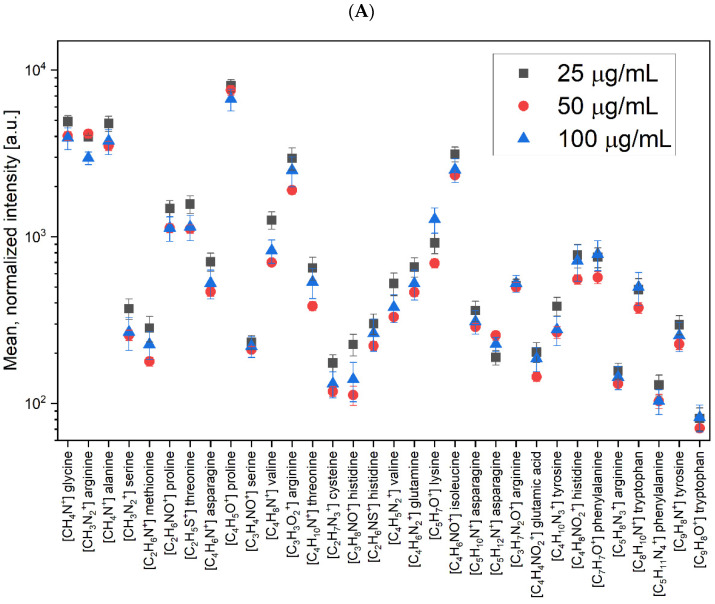

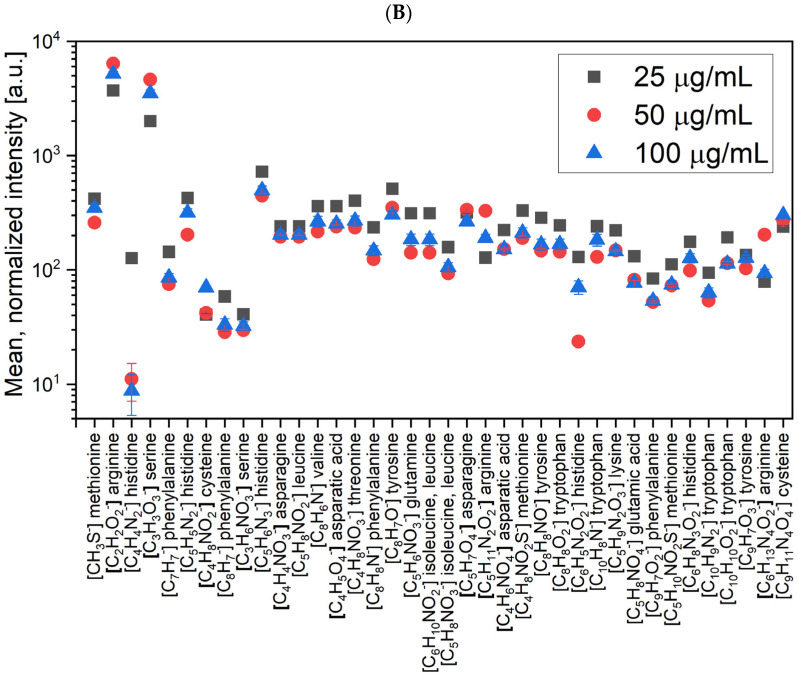
Values of the mean normalized intensities for the characteristic amino acid peaks in (**A**) positive and (**B**) negative ions for the three concentrations of LACT deposited on the surface of functionalized silicon with APTES and GA.

In the first stage of the comparative analysis, carried out using the spectra obtained from ToF-SIMS, the focus was on the peaks characteristic of amino acids. Figure 5 summarizes the mean values of the normalized intensities for the three LACT concentrations. The list of characteristic peaks of amino acids was constructed based on data from the literature and the library of the SurfaceLab program [29,30].

As a results of the analysis shown in Figure 5A, in terms of APTES + GA, for most of the characteristic peaks of the LACT protein, the highest intensity values in the positive polarization were observed at the concentration of 25 µg/mL. Different results were observed for the two ions [CH_3_N_2_]^+^ (arginine) and [C_5_H_12_N]^+^ (asparaginine), where the highest intensities were obtained at the concentration of 50 µg/mL. Only for the characteristic peak of lysine ([C_5_H_7_O]^+^), the highest value was observed at the concentration of 100 µg/mL. Reports from other studies have shown that the effectiveness of sensory surfaces is influenced by the surface density of receptors and the orientation of the capture proteins immobilized on the surface. The sensitivity of chemical reactions depends on the presentation of the binding molecule, and optimal efficiency requires the orientation of the binding centers toward the solution phase [10,31].

A similar analysis was performed for measurements carried out in negative polarization. Figure 5B shows a plot of the mean normalized intensities for all characteristic ions for the LACT protein, for which the one-way ANOVA analysis showed statistically significant differences between the results for the three concentrations considered. For 38 masses corresponding to the characteristic ions in the negative polarization, in most cases, similarly to the positive polarity, higher intensities were obtained at the lowest concentration (25 µg/mL). The trend was different only for seven of the presented masses where, for [C_2_H_2_O_2_]^−^ (arginine), [C_3_H_3_O_3_]^−^ (serine), [C_5_H_7_O_4_]^−^ (asparagine), [C_5_H_11_N_2_O_2_]^−^ (arginine), and [C_6_H_13_N_4_O_2_]^−^ (arginine), higher intensities were obtained for 50 µg/mL while, for the remaining ions characteristic of cysteine (i.e., [C_4_H_8_NO_2_]^−^ and [C_9_H_11_N_4_O_4_]^−^), the highest values were obtained for 100 µg/mL. The results for this ionic polarization were consistent with the results obtained for the positive polarization as, in both cases, for a significant number of characteristic peaks, the highest intensities were found for the lowest concentration. This may mean that the LACT protein, composed of the analyzed amino acids, most effectively binds to the previously functionalized APTES/GA silicon surface at a concentration of 25 µg/mL.

**Figure 6:**
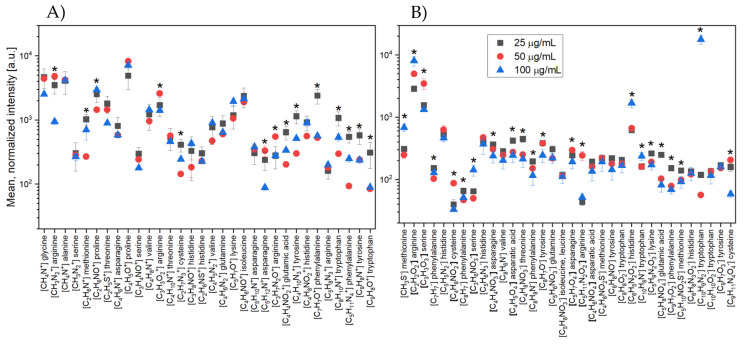
Values of the mean normalized intensities for the characteristic amino acid peaks in the case of (**A**) positive and (**B**) negative ions for the three concentrations of LACT deposited on the surface of functionalized silicon with GOPS. The index “*” means a statistically significant difference at the level of *p* = 0.05 for the studied groups.

The same analysis was performed for the GOPS silane functionalized surfaces (Figure 6).

The graph in Figure 6A shows the mean normalized intensities for most of the characteristic peaks (in positive polarity) of amino acids. For the ions for which a statistically significant difference was found between the intensities for the three selected concentrations of the GOPS silane-functionalized silicon substrate, the designation “*” was assigned. For positively charged ions, statistically significant differences were found for 14 out of 32 masses placed on the diagram; in most cases, the highest intensities were obtained for the lowest concentration (25 µg/mL). The results obtained for the negative polarization confirmed the relationships found using the positive polarization. 

The graph in Figure 6B shows that, in terms of statistically significant differences, the highest intensities were found at a LACT protein concentration of 25 µg/mL. For this polarization, significantly more masses were obtained (22 out of 34 masses), for which the differences between the intensities were statistically significant at the level of *p* = 0.05. After carrying out the analysis presented above, it can be concluded that, for both surface modifications, the highest peak intensities for the characteristic masses of amino acids were obtained at the lowest protein concentration. However, it should be kept in mind that the choice of the most appropriate surface modification/activation procedure directly depends on the system at our disposal (i.e., the selected surface and the characteristics of the receptor); in this case, the LACT protein.

**Figure 7:**
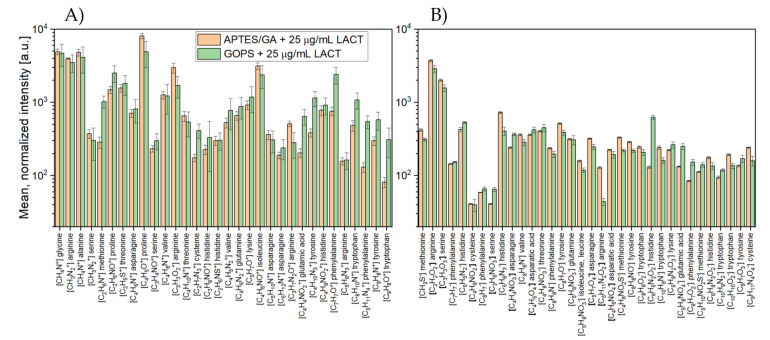
Histogram of characteristic peaks of amino acids in (**A**) positive and (**B**) negative polarity, compared to the used silanes (APTES and GOPS). The data refer to the LACT protein concentration of 25 µg/mL.

In the next step, the obtained results for both silanes (APTES, GOPS) were compared using the data obtained at the protein concentration of 25 µg/mL. The results, in the form of a histogram for the characteristic peaks of amino acids in both polarizations, are shown in Figure 7.

The graph in Figure 7 summarizes the results of the mean normalized intensities for two types of surface functionalization. The first functionalization was based on the use of 3-aminopropyltriethoxysilane (APTES) with glutaraldehyde as a difunctional reagent, and the second with the use of 3-glycidyloxypropyltrimethoxysilane (GOPS). Figure 7A shows the results for 32 masses where, for 11 masses, higher intensities were obtained with the APTES functionalization. In the case of negative polarization, 34 masses were included in the analysis, in which case higher APTES intensities were noted for 18 masses.

Based on the data presented in Figure 7, we could not clearly determine which of the two immobilization methods was more effective. LACT covalent bonding for modified APTES substrates allowed us to obtain comparable results, as no significant difference was recorded for the mean normalized peak intensities characteristic of this group of biomolecules. In turn, the immobilization of proteins on GOPS-activated surfaces, regulated mainly through covalent bonding by free amino groups of proteins, is equally effective [32].

Binding of uEVs

**Figure 8:**
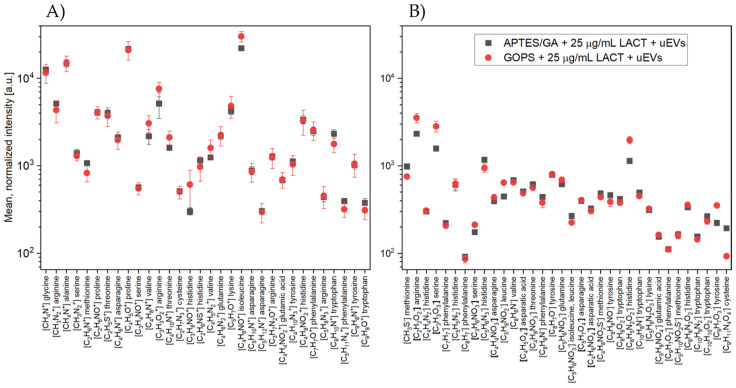
Values of mean normalized intensities for characteristic peaks of amino acids in (**A**) positive and (**B**) negative polarity, compared to the used silanes (APTES and GOPS), with uEVs applied to properly functionalized surfaces at a concentration of 3 × 10^9^ particles/mL. These data refer to the LACT protein concentration of 25 µg/mL.

In the last stage of the experiment, uEVs were applied to properly functionalized surfaces at a concentration of 3 × 10^9^ particles/mL. Figure 8 shows the results comparing the mean normalized intensities for the silicon surfaces modified with two different methods (APTES, GOPS).

These results refer to the LACT protein concentration of 25 µg/mL. The comparative analysis of the spectra was based on the characteristic peaks of amino acids (as previously used) and on the number of peaks characteristic of six lipid groups: fatty acids, glycolipids, glycerophospholipids, sphingolipids, prenols, and sterols.

For both measured polarizations (Figure 8A,B), the presented results indicated that the mean normalized intensities for the peaks characteristic of amino acids for both type of silane-functionalized surfaces (APTES and GOPS) were at a comparable level. Many studies to date have shown that the use of aminopropylalkoxysilanes as coupling agents in biosensors enables better surface bonding, due to the bifunctional nature of these compounds [31,33]. This directly results from the presence of amine groups that catalyze the surface reactions to form siloxane bonds [34]. The structure of γ-aminopropylsiloxane has been previously shown to be inherently more reactive than other aminoalkylsiloxanes, mainly due to their ability to form stable cyclic intermediates. In the case of the two examined surfaces, no significantly higher intensities for the characteristic peaks of amino acids were observed. 

**Figure 9:**
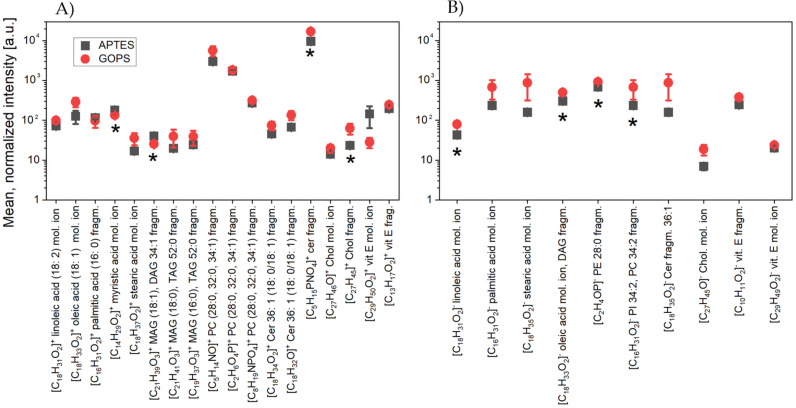
Values of mean normalized intensities for characteristic peaks of lipids in (**A**) positive and (**B**) negative polarity, compared to the used silanes (APTES and GOPS), with uEVs applied to properly functionalized surfaces at a concentration of 3 × 10^9^ particles/mL. These data refer to the LACT protein concentration of 25 µg/mL. The index “*” means a statistically significant difference at the level of *p* = 0.05 for the studied groups.

Figure 9 summarizes the mean normalized intensities for the characteristic peaks of selected lipids belonging to the six lipid groups. In the case of Figure 9A, where the results for positive polarization were placed, the comparative analysis concerned 18 masses. Of these masses, one-way ANOVA showed statistically significant differences in intensities for only four measurement points. For the molecular ion myristic acid [C_14_H_29_O_2_]^+^ (*m/z* 229.02), the MAG and DAG fragment [C_21_H_39_O_3_]^+^ (*m/z* 339.16), and the cholesterol fragment [C_27_H_45_]^+^ (*m/z* 369.37), higher intensity values were obtained for the GOPS-silane surface. As can be deduced, these differences were insignificant, and their number in relation to the quantity of the analyzed masses was insignificant. Therefore, it can be concluded that the intensity values were at similar levels, which would indicate that the number of uEVs bound on both surfaces is at the same level. 

Similar conclusions can be drawn by analyzing the data for negative polarization (Figure 9B) where, for four out of ten analyzed masses, statistically significant differences between the mean intensity values were obtained. For all four masses, corresponding to the molecular ion of linoleic acid [C_18_H_31_O_2_]^−^ (*m/z* 279.24), the molecular ion of oleic acid [C_18_H_33_O_2_]^−^ (*m/z* 281.26), the fragment of phosphatidylethanolamine [C_2_H_4_OP]^−^ (*m/z* 122.92) and the fragment of phosphatidylinotisole [C_16_H_31_O_2_]^−^ (*m/z* 255.24), higher intensities were observed for the surface modified with GOPS. For the remaining masses, the comparative analysis did not show any significant differences. 

## 3. Materials and Methods

### 3.1. Materials and Chemicals

Materials and reagents: Silicon wafers (cat. no. 647780, Sigma Aldrich, St. Louis, MO, USA); 99% ethanol absolute (cat. no. 396480111, POCH); toluene (cat. no. 244511, Sigma Aldrich); chloroform (cat. no. 234431116, POCH); glutaraldehyde (cat. no. 424610237, Chempur, Karlsruhe, Germany); PBS (cat. no. P4417, Sigma Aldrich); human lactadherin (LACT; cat. No. 10853-H088 LC11NO 2901, Sino Biological Inc.), [(3-aminopropyl)triethoxysilane]; APTES (cat. no. 440140, Sigma Aldrich), [(3-glycidyloxypropyl)trimethoxysilane]; GOPS (cat. no. 440167, Sigma Aldrich); bovine serum albumin (BSA; cat. no. 05470, Sigma Aldrich); and cacodylic buffer (Cat. number C4945, Aldrich, St. Louis, MO, USA).

### 3.2. Isolation of uEVs

The uEV samples were isolated from a first-donated specimen (50 mL) collected from the midstream urine of a control donor. The study was conducted according to the guidelines of the Declaration of Helsinki, and approved by the Jagiellonian University Bioethics Committee (permission no. 1072.6120.268.2018, 25 October 2018). Informed consent was obtained from a control donor involved in the study. First, urine sample was centrifuged at 2000× *g* for 30 min at room temperature (RT), in order to remove bacteria, cell debris, and the majority of Tamm–Horsfall protein aggregates [35]. After the centrifugation step, the supernatant was collected and used for the Low-Vacuum Filtration (L-VF) method [36]. In this method, the urine sample was purified and concentrated using a 1000 kDa (MWCO) dialysis membrane, and a low vacuum (−0.3 Bar) was applied. Following the L-VF procedure, the uEV sample was ultracentrifuged for 1.5 h at 150,000× *g* and 4 °C to receive a pellet, which was suspended in 100 µL of deionized water.

### 3.3. Surface Preparation

#### 3.3.1. Modification of Silicon Substrates

**Figure 10:**
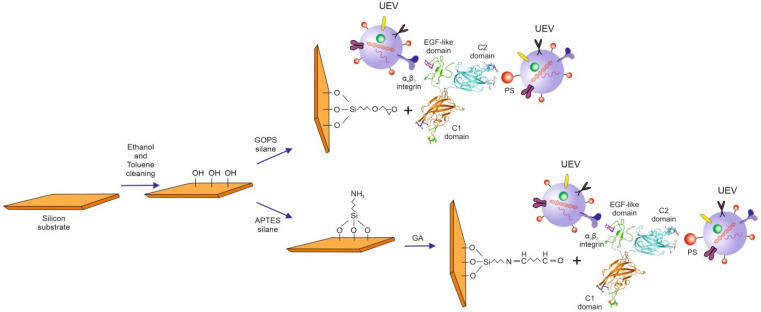
Schematic illustration of the experiment, which investigated the effect of different silanes on urine extracellular vesicles (uEVs) binding by lactadherin (LACT). The LACT model, consisting of an epidermal growth factor (EGF-like) and a C1 domain, was generated using homology modeling with Phyre2 [38]. The C2 domain model corresponds to the crystal structure of the bovine LACT C2 domain (PDB code: 3BN6). The 3D models of each domain were visualized independently in Pymol Molecular Graphics System (ver. 2.3) and placed arbitrarily.

The silicon wafer was functionalized with two different silanes: 3-aminopropyltriethoxysilane (APTES,) which binds to proteins through physical adsorption, and 3-Glycidyloxypropyl)trimethoxysilane (GOPS), which enables biomolecular immobilization through covalent bonding (Figure 10) [37]. These two types of silanes were used to investigate whether the modification of the silane substrate applied in the first step affects the immobilization of LACT and improves the efficiency of uEVs capture.

First, the silicon substrate was cleaned in a toluene and ethanol sequence for 10 min in an ultrasonic bath, then dried under a stream of N_2_. Half of the silicone substrates were then silanized with APTES and half were silanized with GOPS by immersion in a 1% (*v*/*v*) solution in toluene for 10 min, followed by sonication in toluene and ethanol successively, then finally dried in a stream of N_2_ and baked for 20 min at 120 °C [26]. 

APTES substrates were additionally functionalized with GA by immersion in 2.5% glutaraldehyde solution for 20 min. The surfaces were then rinsed with distilled water and dried under a stream of N_2_. The substrates prepared in this way were used to immobilize the active biomolecules.

#### 3.3.2. Immobilization of Biomolecules

Capture of uEVs was performed with LACT immobilized on a silanized surface. To achieve this goal, prepared surfaces were functionalized with LACT by incubation with LACT solutions for 1 h. At this stage, in the second part of the experiment, the influence of LACT concentration on the uEV binding capacity was investigated. For this purpose, we used three different concentrations of LACT (25, 50, and 100 μg/mL), diluted in PBS. 

Finally, the binding of uEVs to the prepared LACT immobilized surface was performed by incubation with the uEV solution suspended in PBS at a volume of 30 µL for one hour, followed by washing with PBS buffer. The final concentration of the uEV solution was 3 × 10^9^ particles/mL. Prior to the measurement by surface techniques, all samples were rinsed with distilled water and dried under a stream of N_2_.

### 3.4. UEVs Characterization

Two methods were used to characterize uEVs: (i) Transmission electron microscopy (TEM), in order to characterize the size and content of uEVs; and (ii) tunable resistive pulse sensing (TRPS), in order to determine the uEVs size distribution from the condensed urine sample.

#### 3.4.1. UEVs Visualization by TEM

For TEM imaging, the uEVs pellet was fixed with 2.5% glutaraldehyde in 0.1 M cacodylic buffer for 2 h at RT. The samples were then post-fixed in 1% osmium tetroxide solution (1 h) and dehydrated by passing through graded ethanol series, then embedded in PolyBed 812 at 68 °C. The ultra-thin sections were collected on 300 mesh grids or one slot made from copper; additionally, the latter was covered with formvar film. A Leica EM UC7 microtome was used to cut the samples. The sections were then contrasted with uranyl acetate and lead citrate. A JEOL JEM 2100HT electron microscope (Jeol Ltd., Tokyo, Japan) was used for observation, at an accelerating voltage of 80 kV.

#### 3.4.2. Characterization of uEV Size Distribution by Tunable Resistive Pulse Sensing 

The uEV population size was measured using qNano Tunable Resistive Pulse Sensing Technology (Izon, Christchurch, New Zealand), according to the manufacturer’s instructions. The isolated uEVs sample was diluted ten times in PBS and measured in triplicate. NP100 nanopores were used, for which the measurement range is 50–350 nm. The nanopore was stretched to 47.12 mm, and a voltage of 0.68 V and pressure of 2 mBa were applied, in order to optimize the nanopore size and the velocity of uEVs passing through the pore. Calibration was performed using CPC100 polystyrene beads with an average size of 100 nm at 1:1000 dilution. The size of the uEVs and sample concentration were determined using the Izon Control Suite software (ver. 3.4).

### 3.5. Surface Characterization

Three physical techniques were used to characterize each step of biomolecule immobilization: (i) Spectroscopic ellipsometry (SE), to calculate the thickness of each molecular layer formed on the surface after each modification step; (ii) atomic force microscopy (AFM), to observe surface topography and calculate surface roughness; and (iii) ToF-SIMS, for the detection of LACT molecules on the prepared surface.

#### 3.5.1. Spectroscopic Ellipsometry

SE is a real-time and non-invasive technique. It was used to assess the thickness of the molecular layer formed on the silicon substrate after each subsequent preparation step. Measurements were made using an M-2000 ellipsometer (Woollam Co., Inc., Lincoln, NE, USA). The spectrum was recorded for two angles, Ψ and Δ, depending on the amplitude and phase difference between the parallel and perpendicular components of the polarized light beam, after reflection from the surface. The wavelength range of 320–700 nm with a constant angle of incidence of 70° was used for measurements. The Cauchy dispersion model was used, which describes the refractive index (n) as a function of the wavelength $\left(\lambda \right)$ [39]

$$n=A+\frac{B}{\lambda 2}+\frac{C}{\lambda 4}.$$

The most accurate fit of the theoretical model to the experimental data was obtained with a refractive index of 1.42 and extinction coefficients equal to 0 and 0.01 for the silane and glutaraldehyde surface, respectively. For the protein layers, the best matches were obtained for a refractive index of 1.45 and an extinction coefficient equal to 0 (zero). Three measurements were made for each sample, and the mean value and standard deviation were obtained. The Complete EASE Data Analysis Manual vs. 3.65 software was used to analyze the obtained data. 

#### 3.5.2. Atomic Force Microscopy

The AFM of the studied surfaces was performed using an Agilent (Santa Clara, CA, USA) 5500 microscope operating in non-contact mode. An AFM tip with constant elasticity of 2 N/m, small-tip radius of <7 nm, and resonance frequency of about 70 kHZ was used, and all measurements were performed at room temperature. Parameters, such as setpoint and gains, were adjusted to ensure minimal noise and a clear image of the examined surface. Images with a scan size of 2 × 2 μm^2^ were collected, with a scan frequency of approximately 1 Hz and a line resolution of 512 × 512. The surface roughness was determined by calculating the root mean square value (Sq RMS), defined as the root mean square value of ordinate values within the definition area, which is equivalent to the standard deviation of heights. This means that the average height distribution of the entire measured AFM image was considered (measured area). When collecting topographic AFM micrographs, the places on the available surface were selected to be in the center of the sample, where all the layers had been applied.

#### 3.5.3. Time-of-Flight Secondary Ion Mass Spectrometry

All measurements were performed using secondary ion mass spectrometry with a ToF-SIMS 5 (ION-TOF GmbH, Münster, Germany) located at the Institute of Physics of the Jagiellonian University. The LMIG Bi_3_^+^ bismuth gun was used as the primary ion source. Primary ions bombarded the tested surfaces with energy of 30 keV at an angle of 45° to the sample surface, and 900 amu was the upper limit of the analyzed mass range. During the measurement, the residual gas pressure in the spectrometer chamber was kept at 10^−7^ mbar. The surface composition analysis was performed with a beam current equal to 0.77 pA, the total dose disposed on the surface was 4.7 × 10^8^, and the total dose density per cm^2^ was 1.88 × 10^11^, which means that the measurement was performed in static mode. These parameters allowed us to obtain detailed information about the chemical composition of the surface of the tested material, while not exposing the surface to damage. In order to neutralize the charge compensation generated on the sample surface, a low-energy electron flood gun was used in the interval between two pulses originating from the primary ion source. The experiments were carried out at the same time and under the same conditions.

Owing to the measurements, spectra with high mass resolution were measured, with a minimum ratio (m/Δm) > 5000 for C_4_H_5_^+^ and C_4_H^−^ peaks, and each obtained spectrum was normalized to the total number of counts during the preliminary analysis. The calibration of each measured spectrum was performed using signals identified from the following positive ions: $H^{+}$, $H_{2}^{+}$, ${\mathrm{CH}}^{+}$, ${\mathrm{CH}}_{2}^{+}$, ${\mathrm{CH}}_{3}^{+}$, and $C_{3}H_{2}^{+}$. Those in the case of negatively charged ions were $C^{-}$, ${\mathrm{CH}}^{-}$, ${\mathrm{CH}}_{2}^{-}$, $C_{2}^{-}$, and $C_{2}H^{-}$. 

For the analysis of ToF-SIMS samples, a holder was used, in which all measured samples were placed simultaneously (two from each tested measurement group). In the first step, a surface analysis was performed over a 500 × 500 µm^2^ area, for negatively and positively charged secondary ions. After measurements were made on the primary surfaces on the basis of emission images obtained for prenols and sterols, secondary surfaces of size 50 × 50 µm^2^ were selected (ROI, Region of Interest), from which the spectra used for the comparative analysis were obtained. A statistical analysis of the obtained results was performed, in order to determine the mean values and standard deviations for a given group, and one-way ANOVA was carried out. Tukey’s test was used to determine the significance of differences between the mean values for both groups. The significance level for the tests was *p* = 0.05. OriginPro 2021 vs. 9.8.0.200 (Academic) software was used to perform statistical analysis and to draw all presented graphs. 

## 4. Conclusions

The aim of the study was to present the effects of different preparations of a silicon surface on the binding of uEVs. We examined two aspects affecting the quality of EV surface bonding. The first concerned the effect of concentration of the lactadherin protein solution, as a biomolecule binding EVs to the surface. The second part investigated how the selection of the surface-modifying silane affected the binding efficiency of uEVs. 

Two techniques—TEM and qNano—were used to characterize the urinary extracellular vesicles. TEM analysis indicated the presence and integrity of uEVs in the sample after the performed isolation. At the same time, owing to the TRPS method, it was estimated that the size of the objects was in the range of 100–300 nm, which proved the presence of medium and large EVs. Three physical methods were used to assess the quality of surface functionalization: ToF-SIMS, AFM, and SE. In the procedure of preparing the silicon surface for capturing biomolecules, each step should be properly planned, and the entire protocol should be designed to optimize the function of describing the end-product. Note that there is no universal recipe and, so, the system should be checked with respect to the molecule we are interested in, as well as taking into account the available methods, while maintaining the lowest possible costs. Immobilized EVs can be subjected to label-free analyses by means ellipsometry, AFM and TOF-SIMS.

The analysis showed that, of the protein concentrations used, the highest intensities for the peaks characteristic of amino acids were obtained with the lowest applied concentration of 25 µg/mL. This is a satisfactory result from the biosensor cost point of view, as the LACT protein used is an expensive point in the sensor preparation procedure. The studies did not show significant differences in the intensity of the characteristic peaks of amino acids and lipids with the two used silanes (i.e., APTES and GOPS) functionalizing the surface. Meanwhile, it is worth noting that preparation of the silicon substrate with the use of GOPS silane required only one preparation step, while APTES + GA required two (including the application of glutaraldehyde). This may be an aspect that supports the use of GOPS. Moreover, after the analysis, it is worth noting the increasing usefulness of the ToF-SIMS technique in the comparative analysis of biological samples.

## Data Availability

Not applicable.
